# Evaluation Methods for Assessing Users’ Psychological Experiences of Web-Based Psychosocial Interventions: A Systematic Review

**DOI:** 10.2196/jmir.5455

**Published:** 2016-06-30

**Authors:** Jacqueline Susan Feather, Moira Howson, Linda Ritchie, Philip D Carter, David Tudor Parry, Jane Koziol-McLain

**Affiliations:** ^1^ Centre for Interdisciplinary Trauma Research Department of Psychology Auckland University of Technology Auckland New Zealand; ^2^ Auckland University of Technology Auckland New Zealand; ^3^ Auckland University of Technology School of Computer and Mathematical Sciences Auckland New Zealand; ^4^ Auckland University of Technology Department of Computer Science Auckland New Zealand; ^5^ Centre for Interdisciplinary Trauma Research School of Clinical Sciences Auckland University of Technology Auckland New Zealand

**Keywords:** eHealth, medical informatics applications, web browser, Web-based, usability, computer systems, psychology, clinical, usability testing, eHealth evaluation

## Abstract

**Background:**

The use of Web-based interventions to deliver mental health and behavior change programs is increasingly popular. They are cost-effective, accessible, and generally effective. Often these interventions concern psychologically sensitive and challenging issues, such as depression or anxiety. The process by which a person receives and experiences therapy is important to understanding therapeutic process and outcomes. While the experience of the patient or client in traditional face-to-face therapy has been evaluated in a number of ways, there appeared to be a gap in the evaluation of patient experiences of therapeutic interventions delivered online. Evaluation of Web-based artifacts has focused either on evaluation of experience from a computer Web-design perspective through usability testing or on evaluation of treatment effectiveness. Neither of these methods focuses on the psychological experience of the person while engaged in the therapeutic process.

**Objective:**

This study aimed to investigate what methods, if any, have been used to evaluate the in situ psychological experience of users of Web-based self-help psychosocial interventions.

**Methods:**

A systematic literature review was undertaken of interdisciplinary databases with a focus on health and computer sciences. Studies that met a predetermined search protocol were included.

**Results:**

Among 21 studies identified that examined psychological experience of the user, only 1 study collected user experience in situ. The most common method of understanding users’ experience was through semistructured interviews conducted posttreatment or questionnaires administrated at the end of an intervention session. The questionnaires were usually based on standardized tools used to assess user experience with traditional face-to-face treatment.

**Conclusions:**

There is a lack of methods specified in the literature to evaluate the interface between Web-based mental health or behavior change artifacts and users. Main limitations in the research were the nascency of the topic and cross-disciplinary nature of the field. There is a need to develop and deliver methods of understanding users’ psychological experiences while using an intervention.

## Introduction

### Internet-Delivered Health Care

The past 15 years have included a burgeoning in the development of Internet-delivered health care. The term eHealth has been widely adopted to describe the application of Internet and communication technology to improve the operation of the health care system and health care delivery [1]. The rationale for offering health services online is similar to the provision of any service online, be it commerce, education, or entertainment. It is accessible anytime and anywhere to an almost unlimited audience and is often cheaper than face-to-face [1]. Within health care, the growth of technology means that eHealth is seen as a means of providing targeted treatment to a wide client base [2,3], and self-help Web-based interventions can also contribute to the reduction of disparities in health care internationally [4,5]. It also allows for a focus on patient-centered customized care “providing the right information, to the right person, at the right time” [6]. Moreover, people now expect to be able to use the Internet to assist in most aspects of their lives, including health care.

The range of health services offered over the Internet encompasses not only physical concerns, such as high blood pressure or diabetes, but also psychological issues such as depression, addiction, and anxiety [7-9]. The provision of eHealth for psychological issues can take many different forms. They include (1) direct human interaction, known as e-counseling or e-therapy, which involve electronic communication (via email, Skype, or messaging for example) between a clinician and client; (2) Web-based psychological interventions such as w the new Zealand government’s depression support site[10] or Sleepio [11], which have information, health assessment screening, and treatment and are targeted to specific issues; (3) Internet-based therapeutic software programs such as gaming and virtual worlds; (4) mobile apps; and (5) other activities such as support discussion groups, blogs, and podcasts [3,12,13].

It makes sense to assume that the proliferation of Web-based psychological interventions could not have occurred without strong supporting evidence, yet there is a notable gap between what is proposed by eHealth interventions and what is actually delivered [14]. As such, the continued investment in, and adoption of, Web-based interventions requires more evaluative information to ensure resources are not wasted on ineffective interventions and that the “best practices of successful programs are rapidly disseminated” [15]. Evaluative information should be relevant to policy makers, health care providers, practitioners, and clients [16,17]. This requires a multifaceted approach, coalescing aspects of traditional psychological intervention evaluations such as efficacy, effectiveness, and measures of the therapeutic relationship, client variables, and therapist feedback, with computer science developed usability testing approaches to gather an overall measure of an intervention’s utility.

### Evaluating Web-Based Psychosocial Interventions

It makes sense that evaluation of a Web-based eHealth intervention should naturally follow much of the same evaluative process as a traditional intervention [18]. Effectiveness can be measured by pre- and postintervention assessments using clinical trials. However, assessments can also be made of the components of the intervention. In traditional face-to-face treatment, this would include the therapeutic relationship and session outcome measures, which gather information on the experience of the user during as well as after the intervention. In eHealth, user experience is often focused on usability testing that generally measures the degree to which the intervention is understandable and easy to use.

#### Effectiveness of Web-Based Psychosocial Interventions

Effectiveness refers to how well a health or psychological treatment works in a real-world setting [19]. Methods used for clinical trials of pharmacological treatments can be applied to complex nonpharmacological interventions such as eHealth initiatives [20]. Randomized clinical trials (RCTs) have found that Web-based interventions are effective in treating a range of psychological issues. For example, individually tailored Internet-delivered cognitive behavioral therapy (CBT) has been found to be an effective and cost-effective means of treating patients with anxiety disorders [9]. A recent meta-analysis of RCTs investigating the effectiveness or efficacy of Web-based psychological interventions for depression found positive results across diverse settings and populations [21]. However, as Richards and Richardson [21] note, effectiveness is only one part, albeit an important one, of an intervention that should be evaluated. For example, effectiveness research does not necessarily investigate the influence of therapist factors.

#### User Experience of Web-Based Psychosocial Interventions

In evaluation research of face-to-face psychosocial interventions, findings have shown that an effective therapeutic relationship in and of itself may be enough to provide successful outcomes for clients [22]. Moreover, this correlation between ratings of the therapeutic relationship and outcome seems unaffected by other variables such as outcome measure or type of intervention employed [23]. These variables are client derived, based on their subjective experiences, and can be adjusted for and evaluated throughout the treatment program or therapy session. As a result, the interaction between the client and clinician is fine-tuned and dynamic, and the clinician is able to constantly monitor the therapeutic relationship and make changes accordingly. This is possible in a dynamic person-to-person interaction but it is not possible in a one-way interaction between a person and a computer.

In the person-computer interaction, the psychological experience of the person using the therapeutic intervention is lost and cannot be responded or adjusted to (although there are developments in this area [24]). This raises three key concerns. First is the potential loss of the therapeutic relationship. Second, the psychological experience of the person may be important to determining usage and adherence to an intervention with some reports of dropout rates exceeding 98% from open-access websites dealing with psychosocial issues [25]. Third, it may be important in understanding issues of psychological safety and well-being for “if information is too complex to understand, especially under periods of duress or high cognitive load” then the intervention may not be as effective [6]. Therefore, the in situ psychological experience of the user may be an important variable to be considered; however, it is not clear how it has been or should be evaluated.

### Methods of Evaluating Web-Based Psychosocial Interventions

#### User-Focused Evaluations

In traditional face-to-face interventions, evaluation of user experience is relatively straightforward. For starters, the client can provide instant and direct feedback to the therapist during a session. In addition, there are a number of psychometrically reliable and valid standardized tools such as the Helping Alliance Questionnaire [26] or the Outcome Rating Scale and Session Rating Scale [27], which directly address user experience. In e-therapy or e-counseling, when there is still a person-to-person relationship via Skype or email, the measurement of the therapeutic process is similar as only the delivery differs [12]. However, when the intervention is provided by a website and is driven by computer-programmed algorithms and automated responses it is much more complex. A cross-disciplinary approach is needed that links the psychosocial approach with user-centered design from computer science [6]. One of the most common ways of evaluating the relationship the user has with a website or Web-based intervention is through usability testing.

#### Intervention-Focused Evaluations

The literature on human-computer interaction yields a number of different methods of evaluating a website, be it a psychosocial intervention or an web-based retail store. One of the most widely used and recognized means of addressing this is usability evaluation [28]. Usability testing is a means of evaluating the design and functionality elements of a website as they apply to the user. However, the end user is not always involved and, if so, any information capturing the user experience is designed to enhance the performance of the intervention in terms of content, design, and navigability of the website, rather than an investigation of the relationship between the user and computer.

Usability inquiry on the other hand is designed to gather broader and more subjective information from the user with regard to the website or intervention. Zhang [29] defined usability inquiry quite broadly as gaining “information about users’ experience with the system by talking to them, observing them using the system in real work (not for the purpose of usability testing), or letting them answer questions (verbally or in written form)” [29]. Therefore, although usability testing provides output from the person using the system, the focus is on functionality of the website. In usability inquiry, there is a subtle shift to understanding the users’ goals, context, profile, feelings, and thoughts during the process of interaction. The output extends beyond issues of website design and functionality to broader concerns of purpose and context [28].

The methods of ethnographic interviewing, contextual inquiry, cognitive interviewing, and situated co-inquiry lend themselves, somewhat naturally, to an exploration of how users may experience a Web-based psychological intervention. They all place the user as the most important piece of the process. Thus, it fits the paradigm of interpretative and participatory research with elements of phenomenological inquiry to gain an understanding of the users’ experience in a natural setting. In some ways, this would come close to understanding the nuance of the relationship between a user and an intervention in a similar way that an evaluation of the therapeutic relationship captures a client’s perception and experience of traditional therapy. However, it is unclear to what extent usability inquiry, or any other methods, have been used to address the psychological experiences of those using Web-based psychosocial interventions. Thus, a systematic literature review was undertaken to assess what methods, if any, have been applied to understanding users’ in situ *psychological* experience with Web-based interventions.

## Methods

### Systematic Literature Review Methodology

A systematic literature review was employed to determine how end users’ psychological experiences of Web-based interventions have been evaluated. A systematic review is beneficial for this relatively new topic for “identifying gaps and weaknesses in the evidence base and increasing access to credible knowledge” [30,31]. The AMSTAR (Assessment of Multiple Systematic Reviews) was selected [31] to guide the review. AMSTAR factors and how they were applied in this study are outlined below. The research question under investigation was defined as “What evaluation methods have been used to explore end-users’ in-situ psychological experiences with web-based psychosocial interventions?”

#### Inclusion and Exclusion Criteria

There were 4 criteria components for selecting studies. First, the *health issues* of interest were those with a psychosocial component for which a person may have been reasonably expected to see a clinician if the Web-based intervention was not available; for example, helping people with common mental health concerns such as stress, anxiety, and depression. Also included were psychosocial or behavior change intervention programs associated with physical ailments, such as rehabilitation and psychological well-being following surgery or living with human immunodeficiency virus infection. The psychosocial element was required to underscore the importance of the user’s psychological experience with the intervention. Thus, interventions with a purely physical component such as smoking cessation were excluded.

Second, the *type of interventions* included were Web-based interventions with a primarily self-help basis that required users to work their way through a series of steps. Excluded were e-counseling and e-therapy programs where the treatment was based on person-to-person interaction rather than a person-to-computer interaction. They were also excluded if they were solely psychoeducation or health promotion websites that did not require an interactive, self-guided program. Online tools (such as “Facebook”) that may be used as part of social skills therapy but not designed for the purpose of treatment or psychosocial intervention were excluded.

Third, the *method* of evaluation had to involve end users’ assessment of their experience, rather than expert assessments. Finally, the *focus* of the evaluation must be the end users’ psychological experience interacting with the intervention. Studies that looked at the overall efficacy or treatment outcome of a Web-based intervention were excluded. Usability studies were excluded if they focused solely on functional Web-development issues (content, layout, design), rather than on the psychological experience of the user. For example, “think aloud” (also called talk aloud or cognitive interviewing) studies that did not elicit psychological insight or experience from the user but discussed use of menu options, friendliness, and ease of use were not included. Basically, the study had to be user focused, rather than intervention or computer focused. Expert usability and heuristic studies were also not of value as they did not address the actual experience of the intended user of the intervention. If an evaluation of the users’ psychological experience formed part of a broader study on the effectiveness study or feasibility study, it was included. As a result of a scoping exercise, we expanded the search protocol to include end users’ psychological experience per se, whether in situ or postintervention.

#### Search Protocol

Keywords applied in the search were determined by an initial review of the literature and modified by feedback from psychologists, computer science usability experts, and a librarian. Sets of terms were proximally searched including experience (“experience,” “evaluation,” “usability”), Internet (“internet,” “web*,” “online,” “eHealth”), health (“mental health,” “behavio*,” “psycho*”), and interventions (“intervention,” “treatment,” “therapy”). To ensure coverage of the health, psychological, and computing components of the topic the following databases were searched: CINAHL (EBSCO), MEDLINE (EBSCO), and Psychology and Behavioral Sciences Collection (EBSCO), Computers and Applied Sciences Complete (EBSCO), ABI/INFORM Global, and IEEE Xplore (see Multimedia Appendix 1). The search was restricted to studies from 2004 to October 2015 because of the relative newness of the field of Web-based interventions. A title scan across non–peer-reviewed journals elicited no studies of interest in the initial scoping, so only scholarly journals were included in the search. A total of 2 studies came from a review of the references of a meta-synthesis of user experience of online computerized therapy for depression and anxiety [32].

#### Search Results

**Figure 1:**
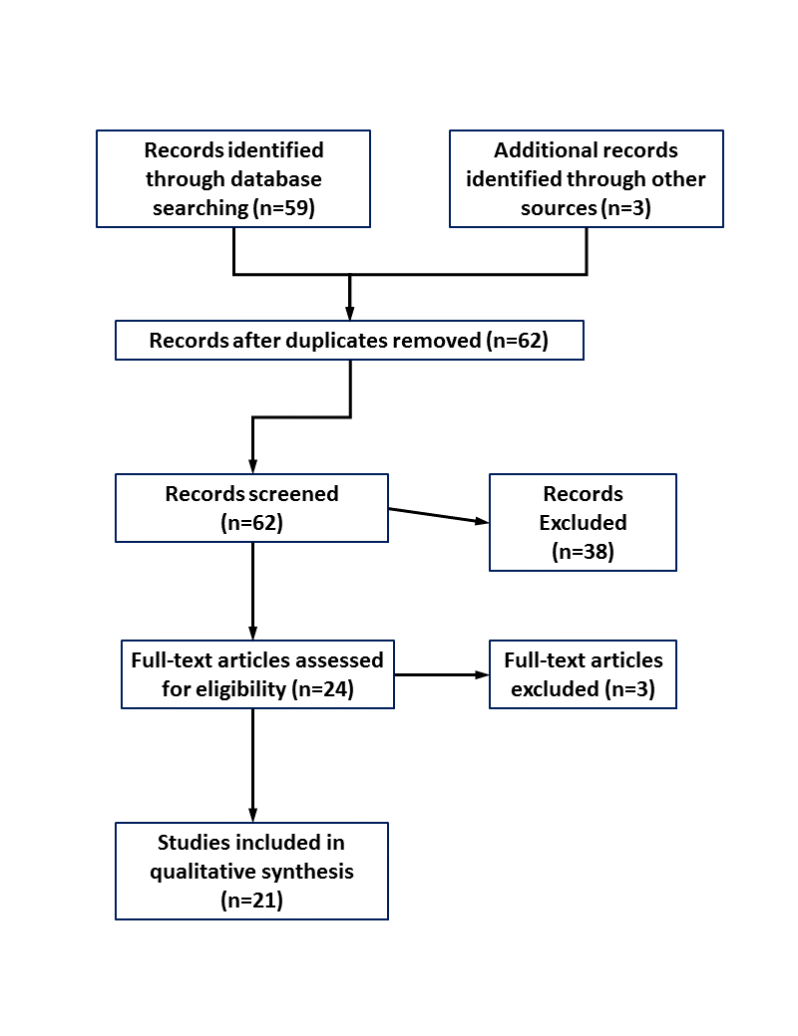
Flow Diagram of Study Selection.

The search protocol resulted in 62 records that were screened by 2 reviewers (LM and LR), with disagreements resolved by the research team (see Figure 1). After final full-text review, 21 studies were identified as meeting the 4 inclusion criteria. Included and excluded studies are listed in Multimedia Appendices 2 and 3, respectively.

### Quality Assessment and Data Synthesis

An assessment of the type and quality of the methods used in the 21 studies was integral to this research project, and screening studies out on the basis of methodological quality would have been counterproductive. Therefore, all the 21 selected studies were included in the synthesis phase. As the identified studies were heterogeneous, a narrative synthesis was undertaken. The a priori data extraction framework involved first, describing the studies (see Multimedia Appendix 2 for study characteristics) and second, their methods of examining user psychological experience of Web-based psychosocial interventions (see Table 1).

**Table 1:** Included studies: methods of examining user psychological experience.

Reference	User experience focus	Methods^a^	Tools	Time of assessment
Baños et al [33]	No	Open-ended questions (online)	Researcher designed questionnaire	Postsession (6 × weekly sessions)
Bendelin et al [34]	Yes	Semistructured interviews (face-to-face)	On the basis of the Client Change Interview	Posttreatment (8-10 months)
Bradley et al [35]	Yes	Semistructured interviews (phone)	Researcher devised based on Theory of Planned Behavior	Posttreatment (1 week)
Cartreine et al [36]	No	Questionnaires (online)	System Usability Scale; Credibility Questionnaire; Assessment of Self-Guided Treatment	Postsession
de Graaf et al [37]	No	Questionnaires (online)	Credibility Expectancy Questionnaire (CEQ); customized questionnaire	CEQ at baseline; questionnaire posttreatment (3 months)
Devi et al [38]	Yes	Semistructured interviews (face-to-face)	Researcher devised interview guide	Posttreatment (6 weeks)
Fergus et al [39]	No	Questionnaire; semistructured interviews (face-to-face)	Treatment Satisfaction Questionnaire; researcher devised interview guide	Posttreatment (time unspecified)
Gega et al [40]	Yes	Questionnaire; semistructured interviews (face-to-face)	Session Evaluation Questionnaire; Session Impacts Scale; Helpful Aspects of Therapy form; Client Change Interview	Questionnaires postsession; Interview posttreatment (time unspecified)
Gerhards et al [41]	Yes	Semistructured interviews (face-to-face)	Researcher devised interview guide	Posttreatment (time unspecified)
Gorlick et al [42]	Yes	Semistructured interviews (phone)	Researcher devised interview guide	Posttreatment (<2 years)
Gulec et al [43]	No	Questionnaires (online)	Researcher designed self-report questionnaires	Online weekly during treatment and posttreatment
Hind et al [44]	Yes	Session evaluation forms; semistructured interviews (phone and face-to-face)	Researcher devised session evaluation forms and interview guide	Evaluation postsession; brief interview postsession 1; interview posttreatment (after completing or withdrawing)
Lara et al [45]	No	Questionnaire (online)	Researcher devised questionnaire	Posttreatment
Lederman et al [46]	No	Semistructured interviews (face-to-face)	Researcher devised interview guide	Posttreatment (time unspecified)
Lillevoll et al [47]	Yes	Semistructured interviews (face-to-face)	Researcher devised interview guide based on phenomenological hermeneutical approach	Posttreatment (time unspecified)
McClay et al [48]	Yes	Semistructured interviews (phone)	Researcher devised interview guide based on motivation, experience, and comparison with other treatments	Posttreatment (time unspecified)
Serowik et al [49]	No	Think aloud usability; questionnaire	Think aloud usability protocol; researcher designed questionnaire; modified Working Alliance Inventory	Usability during session; questionnaire posttreatment or at dropout (time unspecified)
Tonkin-Crine et al [50]	Yes	Unstructured interviews (phone)	Open-ended interview researcher devised	Posttreatment (time unspecified)
Topolovec-Vranic et al [51]	No	Unstructured interview (phone)	Unspecified	Weekly during 6-week program and 12 months postenrollment
Van Voorhees et al [52]	No	Questionnaire; diaries	Researcher designed questionnaire	Diary during and after session; questionnaire posttreatment (time unspecified)
Wade et al [53]	No	Semistructured interviews; survey	Unspecified	At follow-up (time unspecified)

^a^ Other measures may have been used in the study such as pre- and postbaseline measures of diagnosis but these were not included in the data extraction as they did not concern user experience.

## Results

### Overview of Included Studies

#### Intervention Type

The interventions were Web-based self-help interventions consisting of a number of modules (ranging from 3 to 12), most expected to be used on a weekly basis. Although all of the studies were predominantly self-help programs, some were augmented by other elements such as an online diary [37,38,41], social or peer group support in the form of discussion or chat groups [38,39,42,44,46], and some degree of therapist or tailored guidance to support the self-help component [34,38-40,43,53]. A total of 9 studies focused on an intervention in the pilot or development phase [33,34,36,39,43,46,49,52,53], whereas 13 studies focused on existing treatment programs such as “MoodGYM” or “Colour Your Life” [35,37,38,40-42,44,45,47,48,50-52]. All of the interventions contained interactive or homework components and some reported including multimedia such as audio or video in content delivery.

Of the 21 studies, most studies focused on the treatment of psychological issues. A total of 10 studies evaluated interventions designed to treat or prevent the development of depression using principles of CBT as the modality of treatment [34,36,37,40,41,44,45,47,51,52]. One intervention used CBT to assist adolescents with stress, anxiety, and depression [35]. Of the studies, 2 focused on delivering mainstream treatment for eating disorders online [43,48] and 1 on the treatment of first psychotic episodes through the use of CBT, support, and psychoeducation [46]. One intervention used motivational interview principles to support veterans with war exacerbated psychiatric issues apply for jobs and benefits [48]. Another aimed to reduce child behavior problems and parenting stress for young children with traumatic brain injury (TBI) via an online self-guided program and live coaching [53]. A total of 4 studies focused on interventions delivering psychological well-being and support to those with chronic or acute physical issues including irritable bowel syndrome [50], cancer [39,42], and angina [38]. Another study used a positive psychology approach to enhance mood in general populations [33].

#### Study Characteristics

Of the 21 studies, 13 were published from 2012 onward (see Table 1). A total of 10 studies were explicitly focused on the evaluation of the patient or users’ psychological experience of the intervention [34,35,38,40-42,44,47,48,50]. Of the remaining 10 studies, 8 gathered user experience as part of the development and pilot testing of the intervention [36,39,43,45,46,49,52,53]. Another study collected information on user experience to assess the acceptability and use of online CBT for depression [37], and another looked at the efficacy of using an online CBT intervention for depression with patients experiencing depression along with their TBI [51].

### Evaluation of User Experience

#### Rationale for Evaluating User Experience

The rationale for evaluating user experience varied across the studies. Of these, 5 studies were seeking information on the users’ psychological experience of using the website to inform improvements [36,39,45,46,49]. A total of 6 studies were interested in finding out how to increase use of online interventions by understanding barriers to change [35] and acceptability in particular populations [42-44,51,52].

#### Process of Evaluating User Experience

The process of gathering user experience information also varied. A total of 13 studies relied on only one form of evaluation method. Of these, 10 studies used only an interview at the end of the treatment program (with a range of 1 week to 2 years posttreatment, where specified) to ask users about their experience with the intervention. Of the studies, 5 used face-to-face interviews [34,38,41,46,47] and 5 employed phone interviews [35,42,48,50,51]. The interviews were conducted by the researcher or research assistants (when specified) and their duration ranged from 19 minutes [35] to 111 minutes [34]. Of the studies, 3 employed only questionnaires to investigate user experience. Of the 3 studies, 2 deployed it at the end of each treatment session [33,36] and the other [37] at the beginning of treatment and then 3 months posttreatment.

The remaining 8 studies relied on a mixed methods design to investigate user experience. Of these, 4 studies employed a posttreatment semistructured interview, 1 study [39] complimenting it with a posttreatment questionnaire and another study with a posttreatment satisfaction survey [53]; Gega and colleagues [40] with postsession questionnaires and Hind and colleagues [44] with a postsession evaluation form. One study used weekly evaluation questions and a postintervention questionnaire [43]. A further study used a during and after session diary to capture user feedback combined with a posttreatment questionnaire [52] and another used module and a postintervention evaluations along with content analysis of the site’s discussion forums [45]. The final study used a think aloud usability process during the session followed by an end-of-treatment questionnaire [49]. Note that this was the only study employing a usability testing method that included a user experience focus, explicitly asking for feelings during the session, and a questionnaire to elicit user experience with the therapeutic alliance during the Web-based intervention.

#### Tools Used to Evaluate User Experience

Tools used to gather user experience information ranged from customized to off-the-shelf. Of the 14 studies that used some form of interview with users, 12 were semistructured with the topic guide designed by the research team. Of these, 2 studies provided a theoretical basis for the design of the interview guide. Lillevoll and colleagues [47] followed a phenomenological-hermeneutic approach to understand the lived experience of the user of the intervention in the natural setting, and Bradley and colleagues [35] followed a Theory of Planned Behavior. A further 2 studies [34,40] based the interview guide on the Client Change Interview (CCI), which was designed as an empathic exploration of aspects of a client’s experience with traditional face-to-face counseling and therapy.

Questionnaires used to evaluate user experience were either researcher designed or existing tools. Of the studies, 7 used self-customized questionnaire or evaluation forms [33,37,43-45,52,53]. Others relied on questionnaires designed to gain feedback about the therapeutic process, sessions, expectations, and outcomes (Table 2).

**Table 2:** Treatment feedback questionnaires used to evaluate user experience.

Questionnaire	Purpose	Cited in^a^
Credibility Questionnaire	Credibility of computer programs, psychotherapy, and treatment	Cartreine et al [36]
Assessment of Self-guided Therapy	Acceptability of treatment (eg, comfort, personal acceptance)	Cartreine et al [36]
Credibility Expectancy Questionnaire	Expectation and rationale for treatment	de Graaf et al [37]
Treatment Satisfaction Questionnaire	Satisfaction and experience (convenience, quality, value) with online treatment	Fergus et al [39]
Session Evaluation Questionnaire	User experience with the session in terms of depth, positivity, smoothness, and arousal	Gega et al [40]
Session Impact Scale	User view of session impact on understanding, problem solving, therapeutic relationship, and hindering impact	Gega et al [40]
Helpful Aspects of Therapy	Identify helpful or hindering aspects of the treatment session	Gega et al [40]
Working Alliance Inventory	Self-report assessment of user experience of alliance with treatment (modified for online)	Serowik et al [49]

^a^ Further details of questionnaires can be found by consulting the references.

#### Analysis of User Experience Data

As this study is focused on the methods of evaluating user experience as opposed to user experience per se, the results of the studies are not presented in detail. In summary, quantitative analysis was carried out on the statistical data gathered in the questionnaires and descriptive summaries reported where appropriate. The qualitative user experience information in 19 of the studies was analyzed using content analysis to elicit emerging themes. Of these, 2 studies followed elements of grounded theory methodology to develop theories of user experience with Web-based interventions [41,52]. One study took a phenomenological-hermeneutic approach [47].

Overall, these studies reported thematic categories to describe the users’ experiences within the frame of the study purpose. Thus, the themes that evolved were varied and included user experience in terms of the therapeutic process [40,41], individual and social aspects of the intervention [33,40-42,47], as well as Web-based characteristics of the intervention [40-42,48-50]. Process issues included motivation and user profile [34], privacy and help-seeking [35], overall experience [37,46], barriers [50,52], expectations [37], personalization, solitude, and social, individual, and contextual features [40,41]. For example, in one of the grounded theory studies, Gerhards and colleagues [41] identified themes of the user experience in terms of the computer, individual, social, research, and environmental contexts.

## Discussion

### Key Findings

This systematic review identified a gap in examining users’ *psychological* experience of Web-based interventions in situ. Among studies using usability testing, only 1 study [49] explicitly explored what it felt like for the user in-session. Most were focused on the functionality of the intervention in terms of Web design, ease of use, and readability. Usability inquiry methods such as situated co-inquiry proposed by Carter [54] or ethnographic and contextual inquiry discussed by Cooper et al [28] are not yet evident in the literature. It is important that our methods of evaluating Web-based interventions include opportunities for uncovering the relationship between the user and the computer as a social agent. The studies that did focus on the therapeutic experience online tended to evaluate it postsession or posttreatment using tools designed for evaluating face-to-face interventions. For Web-based interventions, as called for by Knowles et al, “it is likely that more in-depth, observational data collection methods will be necessary to better capture user experience in [the] future” (p11) [32].

The rationale for postsession and posttreatment evaluation occurred when the interest was social science rather than computer science and the focus was the treatment experience rather than the mode in which it is delivered. Some of the studies sought to understand the therapeutic process at a session level [40,44], the therapeutic relationship [34,40], and how overall experience translated from traditional delivery channels of treatment into Web-based treatments [41,43]. Other studies aimed to enhance the treatment program and increase usage and acceptability of particular populations to certain interventions, such as adolescents with psychological distress [35] or those with psychosocial issues associated with physical health problems [38,39,42,44,50,51]. Postsession and posttreatment evaluations were also used to understand barriers to usage [52] and motivations to participate in Web-based interventions [37,48]. Most of these evaluations used semistructured interviews or questionnaires to collect information. Posttreatment semistructured interviews (face-to-face or phone based) were the key methods used to understand the experience of the user.

An in-depth review of the methods was not possible because of gaps in the literature on timing, length, interviewer characteristics, and topic guide details. It is evident however that the problem with posttreatment evaluations is the potential time delay between treatment and recollection of experience and this potentially affects validity and reliability of the information. The time between treatment and evaluation varied, some took place 1 week posttreatment [35] and another had a gap of up to 2 years [42]. Some studies did not actually specify how long following treatment that the evaluations occurred. Some interviews were conducted by phone for 20 minutes [48] and others reported almost 2-hour face-to-face interviews [34] and thus the depth and quality of information varied accordingly. In addition, the characteristics of the interviewers were also sometimes overlooked. When specified, the interviews were conducted by the researcher or research assistant who may or may not have had relevant clinical training. In general, the topic guides or questions asked by the researchers were not published so that the areas discussed with the participants are unknown, although it can be extrapolated to some extent by the focus of the research. Some studies provided example topics or questions. For example, Devi and colleagues [38] presented a table of example interview questions that included “What were your initial thoughts and feelings regarding this web-based programme?” and “What was your overall experience of using the programme?” Having more information as to the topics covered would be useful to understand in detail what aspects of user experience were investigated.

In addition to interviews, questionnaires were also used; most commonly they were standardized tools to measure experience with traditional treatment or therapy modified for eHealth. For example, Gega and colleagues [40] used the Session Impact Scale, Session Evaluation Questionnaire, and Helpful Aspects of Therapy questionnaire to gather immediate feedback from users following each online session. This elicited in-depth session and overall feedback that were categorized into a number of themes, such as the experience of “learning by doing” or having no fear of being judged by a Web-based intervention. These questionnaires were designed to understand treatment experience and outcomes and were not concerned with the functionality of the website. The use of the CCI as the basis for the interview guide by Bendelin and colleagues [34] and Gega and colleagues [40] reinforced this focus on treatment versus delivery that was also evident in the analysis for the evaluation findings. The analysis of user feedback employed by these studies reflected an interest in understanding users’ psychological experience for its own sake rather than in relation to the intervention alone. The value of the information elicited was dependent on the purpose of the research, and each of the 21 selected studies had slightly different foci and rationale for exploring the experience of the user.

Most of the studies that used an interview approach used inductive content analysis to interpret the findings in terms of themes and subthemes of experience. Examples of themes included user profile and motivation [34], privacy, and help-seeking [35]; solitariness and personalization [40]; and the individual, social, computer, and wider contexts of experience [41]. Interviews are widely used in information systems research and eHealth evaluation, but it is important to be clear that an interview is an artificial and additional intervention. Myers and Newman’s [55] description of the dramaturgical model as originally described by Goffman [56] may aid interviewers to move between evaluation and clinical issues.

Although functionality was not a driver in the research, themes of intervention characteristics [36,40,41] and Web engagement [47] did emerge. This suggests that users’ psychological experiences are determined in part by the psychological issue and treatment process, and in part by the channel or mode of delivery. In other words, the functionality explored in usability testing might be a piece of the experience but is by no means all of it.

The choice of methods of evaluation depends primarily on which aspect of the intervention is most critical to measure—for example, effectiveness, usability or engagement—and on the maturity of the implementation of the intervention, and multiple methods may be appropriate. For early prototypes, interviews directly after the intervention are appropriate in order to gain an understanding of all of the issues and potential benefits as seen by the users. Larger-scale interventions will be more likely assessed by questionnaires, often modified from existing assessments. However, these should occur as quickly as possible after the intervention so that the users still have the experience fresh in their minds. Standard functional questionnaires may be preferred when Web-based treatment approaches are being compared with other interventions. However, all of these evaluation approaches must bear in mind the “emergent” nature of eHealth interventions and the degree to which the objectives and nature of the evaluation of the intervention may differ as prototypes are developed and evaluators gain experience [57]. The unique role of eHealth interventions also needs to be considered; as these inherently deal with people’s well-being, there is a need for closer evaluation of the psychological aspects of a user’s experience.

Overall, the evaluation of users’ psychological experiences with psychosocial interventions is a new yet growing area. Of the 21 studies in the final selection, only 10 were explicitly focused on understanding users’ psychological experience (as opposed to usability, outcome, or overall evaluation). Of those 10 studies, none were published before 2009 and 6 were published in the past 2 years. The nascency of the topic and exploratory nature of this study means that a number of opportunities for future research and action in the area are possible.

### Methodological Limitations

The methodological limitations of this study derived from the cross-disciplinary nature of the topic, resource constraints, and the newness of the field that could have resulted in missed studies. Searching across databases from the computer science and health and social science fields precluded the use of consistent search protocols in terms of qualifiers and data fields searched. Indeed, as noted by reviewers, several studies were missed in the search process [58-61]. Examining these studies, however, offered no additional methods of user inquiry. For example, the Helpful Aspects of Therapy questionnaire used by Gega et al [40] was also used by Richards and Timulak [61]. In addition, the search process was limited to scholarly material. In hindsight, the resource constraint could have been mediated somewhat by limiting the search date to studies published in the past 5 years.

The retrieved studies were not subjected to a quality review. This was due to the nature of the study that was looking at methods used rather than study outcomes. Overall, one could then argue that the search was semisystematic with the front end (the search process) meeting the requirements of a predetermined and comprehensive search, but the back end (data extraction and synthesis) was less systematized and more exploratory in nature. It reflects the challenges outlined by Jesson et al [62] and Curran et al [30] in conducting systematic reviews across disciplines.

There is a lack of consistency in the terminology used to describe Internet-delivered interventions. An effort to create and broadly ratify agreed key or subject words would make the dissemination of information much easier. “eHealth” is almost as broad a term as “health”—searching for eHealth (or mHealth) interventions is as wide a scope as searching for health interventions. Therefore, as the area grows and consolidates, categorization needs to be clearer and differentiate from other deliveries such as telemedicine. Interventions need to be defined more by type (eg, CBT, mindfulness, psychoeducation) with an agreed identifier such as Web-based, Internet-based, or online for example.

### Future Research and Implications

With regard to Web-based psychosocial interventions, there is a lack of cohesion between computer science literature, focused on the technical design, and health literature, focused on the treatment process and its impact. Combining modes of assessment prevalent in one discipline, such as usability testing from computer science, with session and treatment evaluations from psychology and social sciences could bridge this gap. This collaboration could contribute to the development of best practice protocols for understanding users’ psychological experience that might include an evaluation of the in situ experience of the participant using the system, postsession impact, and a reflective posttreatment review. For example, combining the approach by Serowik et al [49] using a feelings-based think aloud usability and a posttreatment therapeutic evaluation (such as the Working Alliance Inventory or CCI) with the type of session evaluations used by Gega et al [40] would provide a comprehensive view of the user’s experience, as would a user experience–focused usability inquiry method such as situated co-inquiry [54]. Research approaches focused on capturing user experience in situ help us understand the impact of Web-based interventions moment-to-moment, as well as their overall effectiveness. There is a role for clinicians in this process along with computer usability and design experts.

Overall, it is the experience of the user that is important in delivering acceptable and useful treatment. An understanding of user experience including their expectations and responses is essential to increasing acceptability, effectiveness, and adherence to cost-effective, broadly accessible Web-based psychosocial interventions. The therapeutic process is important to treatment and the way it is assessed needs to keep up with the way in which therapy is delivered. As the delivery of health changes, there needs to be increasing collaboration among disciplines. This will contribute not only to robust best practice but also to the creation of new and agreed terminology and a cohesive body of literature to ensure broad and effective dissemination of knowledge. A critical understanding of user experience of eHealth is needed to improve outcomes for people who look to the Internet for help.

## Appendix

Multimedia Appendix 1 Database search details.

Multimedia Appendix 2 Included studies.

Multimedia Appendix 3 Excluded studies.

## Abbreviations

AMSTAR: Assessment of Multiple SysTemAtic Reviews
CBT: cognitive behavioral therapy
CCI: Client Change Interview
RCT: randomized clinical trial
TBI: traumatic brain injury
