# Supplementary material for: Evaluation Methods for Assessing Users’ Psychological Experiences of Web-Based Psychosocial Interventions: A Systematic Review
Source: J Med Internet Res. 2016 Jun 30;18(6):e181. doi: 10.2196/jmir.5455 (PMC4945819; doi:10.2196/jmir.5455)
Supplement: Multimedia Appendix 1 [file jmir_v18i6e181_app1.pdf]

Appendix 1: Database search details

Database	Source	Field	Search terms	Hits	Screened
EBSCO Health Databases (excluding Sports/Dental)	Peer reviewed	All	((online N5 intervention) OR (online N5 treatment) OR (online N5 therapy) OR (web* N5 intervention) OR (web* N5 treatment) OR (web* N5 therapy) OR (internet* N5 intervention) OR (Internet* N5 treatment) OR (internet* N5 therapy)) AND ("mental health" OR psych* OR behavio*) AND (experience OR evaluation OR usability)	764	37
EBSCO Computers & Applied Sciences Complete	Peer reviewed	All	((online N5 intervention) OR (online N5 treatment) OR (online N5 therapy) OR (web* N5 intervention) OR (web* N5 treatment) OR (web* N5 therapy) OR (internet* N5 intervention) OR (Internet* N5 treatment) OR (internet* N5 therapy)) AND ("mental health" OR psych* OR behavio*) AND (experience OR evaluation OR usability)	122	13
EBSCO Health Databases (Exc Sports/Dental)	All	All	(internet AND usability AND ehealth)	31	8
EBSCO Health Databases (Exc Sports/Dental)	All	All	((“cognitive walk through” OR “think aloud”) AND ehealth)	5	1
EBSCO Computers & Applied Sciences Complete	All	All	(internet AND usability AND ehealth)	26	0
EBSCO Computers & Applied Sciences Complete	All	All	((“cognitive walk through” OR “think aloud”) AND ehealth)	3	0
IEEE	Open	Meta-data	(experience AND evaluation AND ehealth AND intervention AND pscyho*)	89	0

ABI Inform	Open	Full text	((online N5 intervention) OR (online N5 treatment) OR (online N5 therapy) OR (web* N5 intervention) OR (web* N5 treatment) OR (web* N5 therapy) OR (internet* N5 intervention) OR (Internet* N5 treatment) OR (internet* N5 therapy)) AND ("mental health" OR psych* OR behavio*) AND (experience OR evaluation OR usability)	346	0
**Total**				**138 6**	**59**

Table Note: Search limited to studies published from 2004-2015; Title and abstracts reviewed for duplication, inclusion and exclusion.
