# Supplementary material for: Evaluation Methods for Assessing Users’ Psychological Experiences of Web-Based Psychosocial Interventions: A Systematic Review
Source: J Med Internet Res. 2016 Jun 30;18(6):e181. doi: 10.2196/jmir.5455 (PMC4945819; doi:10.2196/jmir.5455)
Supplement: Multimedia Appendix 2 [file jmir_v18i6e181_app2.pdf]

Appendix 2. Included studies

Reference	Intervention	Study Objective	User Experience Focus
Baños, et al. (2014) [33]	• EARTH of Well-Being,<br>• 3 self-guided activity modules done over 6 sessions<br>• Promote positive mood and relaxation in users	Development and evaluation	No
Bendelin et al. (2011) [34]	• Pilot CBT website (not specified)<br>• Self-help, some guided therapy<br>• Treatment of depression	Qualitative user experience	Yes
Bradley, Robinson, & Brannen (2012) [35]	• "Feeling Better"<br>• Self-help CBT modules<br>• Adolescents with psychological distress	Acceptability of the programme for adolescents	Yes
Cartreine, Locke, Buckey. Sandoval, & Hegel (2012) [36]	• Internet multimedia problem solving therapy (not specified)<br>• Simulation of 6 clinical sessions<br>• Built for NASA as remote treatment of mild depression	Pilot study	No
de Graaf, Huibers, Riper, Gerhards, & Arntz (2009) [37]	• "Colour Your Life"<br>• Multimedia, interactive, modular self-help and online diary.<br>• Treatment of sub-threshold depression	Use and acceptability	No
Devi, Carpenter, Powell & Singh (2014) [38]	• "Activate you Heart"<br>• Six-week self-help, nurse feedback, chat groups, on-line diary<br>• Cardiac rehabilitation; lifestyle and psychological well-being programme	User experience	Yes
Fergus et al. (2014) [39]	• "Couplelinks"<br>• Six modules, interactive with therapist, chat room<br>• Breast Cancer support for couples	Feasibility and acceptability	No
Gega, Smith & Reynolds (2013) [40]	• "Beating the Blues"<br>• Modular CBT with information, homework, monitoring<br>• Treatment of depression	Experience between CBT on-line and therapist CBT	Yes

Reference	Intervention	Study Objective	User Experience Focus
Gerhards et al. (2011) [41]	• "Colour Your Life"<br>• Multimedia, interactive, modular self-help and online diary.<br>• Treatment of sub-threshold depression	Patient experience	Yes
Gorlick, Bantum, & Owen (2014) [42]	• health-space.net<br>• 12-week interactive self help and support group<br>• Provide social and psychological support to assist cancer patients	Understand experience of those who do not engage fully with the program	Yes
Gulec et al. (2011) [43]	• Internet-based Aftercare for Patients with Eating Disorders “EDINA”<br>• Self-help modules with psychoeducation, forums and individual chat sessions with online counsellors<br>• Maintenance treatment for patients with eating disorders	Program assessment	No
Hind et al. (2010) [44]	• "MoodGYM" and "Beating the Blues"<br>• 8 –week (Beating the Blues) or 6-week (Mood Gym) Self-help CBT modular programme<br>• Treatment of depression in people with chronic disease, (multiple sclerosis).	Acceptability of program for chronic disease patients	Yes
Lara et al. (2014) [45]	• HDep (Help for Depression)<br>• Psychoeducation and seven self-help modules<br>• Complementary treatment for depression	Website development	No
Lederman, Wadley, Gleeson, Bendall, & Alvarez-Jimenez (2014) [46]	• Moderated Online Social Therapy (MOST) (website not specified)<br>• Modular CBT, psychoeducation and Positive Psychology, and moderated support groups (6 week trial)<br>• Support for adults experiencing First Episode Psychosis	Intervention design and evaluation	No

Reference	Intervention	Study Objective	User Experience Focus
Lillevoll et al. (2013) [47]	• "MoodGYM"<br>• Self-help CBT modular programme<br>• Treatment of depression	Patient experience	Yes
McClay, Waters, McHale, Schmidt, & Williams (2013) [48]	• "Overcoming Bulimia Online" (OBO)<br>• Multimedia, interactive, self-help modules<br>• Treatment of Bulimia Nervosa (BN)and eating disorders not otherwise specified (EDNOS) attitudes towards cCBT	Investigate patient experiences	Yes
Serowik, Ablondi, Black, & Rosen (2014) [49]	• Website (not specified)<br>• Multimedia interactive Motivational Interviewing framework in 3-sessions<br>• Assist veterans with psychiatric conditions apply for benefits or work	Website development	No
Tonkin-Crine et al (2013) [50]	• "Regul8"<br>• Self-management via CBT in 8 interactive modules<br>• Irritable Bowel Syndrome management of psychological issues	User experience nested within an RCT study	Yes
Topolovec-Vranic et al. (2010)[51]	• MoodGYM<br>• Internet-based, interactive CBT programme with 6 modules<br>• Treatment for depression, prevent and decrease symptoms	Efficacy of program among persons with traumatic brain injury	No
Van Voorhees et al. (2007) [52]	• Depression prevention programme (not specified)<br>• 11 self-help modules<br>• Prevent depression in young adults (<25 years)	Development and evaluation	No
Wade et al. (2009)[53]	• I-InTERACT<br>• Resources, 10 multimedia, self-guided sessions and 5 supplementary, live parent skills couching - Parent Child Interaction Treatment (PCIT)<br>• Reduce child behaviour problems	Efficacy of program for families with young children with Traumatic Brain Injury	No

Reference	Intervention	Study Objective	User Experience Focus
	and parenting stress for young children with TBI		
