# Supplementary material for: Evaluation Methods for Assessing Users’ Psychological Experiences of Web-Based Psychosocial Interventions: A Systematic Review
Source: J Med Internet Res. 2016 Jun 30;18(6):e181. doi: 10.2196/jmir.5455 (PMC4945819; doi:10.2196/jmir.5455)
Supplement: Multimedia Appendix 3 [file jmir_v18i6e181_app3.pdf]

Appendix 3. Excluded studies

Reference	Reason to exclude
Adams, J., Neville, S., & Dickinson, P. (2013). Evaluation of Bro Online: an Internet-based HIV prevention initiative for gay and bisexual men. International Journal of Health Promotion & Education, 51(5), 239-247. doi: 10.1080/14635240.2012.702502	Intervention type and health issue
Ammann, R., Vandelanotte, C., De Vries, H., & Mummery, W. K. (2013). Can a Website-Delivered Computer-Tailored Physical Activity Intervention Be Acceptable, Usable, and Effective for Older People? Health Education & Behavior, 40(2), 160-170. doi: 10.1177/1090198112461791	Not users' psychological experience and health issue
Atkinson, N. L., Saperstein, S. L., Desmond, S. M., Gold, R. S., Billing, A. S., & Tian, J. (2009). Rural eHealth nutrition education for limited-income families: an iterative and user-centered design approach. Journal of Medical Internet Research, 11(2), e21-e21. doi: 10.2196/jmir.1148	Not users' psychological experience and health issue
Beattie, A., Shaw, A., Kaur, S., & Kessler, D. (2009). Primary-care patients' expectations and experiences of online cognitive behavioural therapy for depression: a qualitative study. Health Expectations, 12(1), 45-59. doi: 10.1111/j.1369-7625.2008.00531.x	Intervention type
Bowen, A. M., L., W. M., M., D. C., & S., C. (2008). Internet based HIV prevention research targeting rural MSM: feasibility, acceptability, and preliminary efficacy. Journal of Behavioral Medicine, 31(6), 463-477. doi: 10.1007/s10865-008-9171-6	Not users' psychological experience and health issue
Carrard, I., Fernandez-Aranda, F., Lam, T., Nevonen, L., Liwowsky, I., Volkart, A. C., . . . Norring, C. (2011). Evaluation of a guided internet self-treatment programme for bulimia nervosa in several European countries. European Eating Disorders Review, 19(2), 138-149. doi: 10.1002/erv.1043	Not users' psychological experience
Carswell, K., McCarthy, O., Murray, E., & Bailey, J. V. (2012). Integrating Psychological Theory Into the Design of an Online Intervention for Sexual Health: The Sexunzipped Website. Journal of Medical Internet Research, 14(6), 11-11. doi: 10.2196/resprot.2114	Not users' psychological experience
Cunningham, J. A., & Van Mierlo, T. (2009). Methodological issues in the evaluation of Internet-based interventions for problem drinking. Drug & Alcohol Review, 28(1), 12-17. doi: 10.1111/j.1465-3362.2008.00001.x	Not users' psychological experience
Currie, S. L., McGrath, P. J., & Day, V. (2010). Development and usability of an online CBT program for symptoms of moderate depression, anxiety, and stress in post-secondary students. Computers in Human Behavior, 26(6), 1419-1426. doi: 10.1016/j.chb.2010.04.020	Not users' psychological Experience
Demment, M. M., Graham, M. L., & Olson, C. M. (2014). How an online intervention to prevent excessive gestational weight gain is used and by whom: a randomized controlled process evaluation. Journal of Medical Internet Research, 16(8), e194-e194. doi: 10.2196/jmir.3483	Not users' psychological Experience

**Reference**	**Reason to exclude**
Duffy, S. A., Fowler, K. E., Flanagan, P. S., Ronis, D. L., Ewing, L. A., Waltje, A. H., & Eysenbach, G. (2013). The Development of the Tobacco Tactics Website. Journal of Medical Internet Research, 15(6), 1-1. doi: 10.2196/resprot.2445	Not users' psychological experience and health issue
Durand, M.-A., Wegwarth, O., Boivin, J., & Elwyn, G. (2012). Design and usability of heuristic-based deliberation tools for women facing amniocentesis. Health Expectations, 15(1), 32-48. doi: 10.1111/j.1369-7625.2010.00651.x	Health issue
Graaf, M. d., Totte, J., Breugem, C., Os-Medendorp, H. v., Pasmans, S., & Eysenbach, G. (2013). Evaluation of the Compliance, Acceptance, and Usability of a Web-Based eHealth Intervention for Parents of Children With Infantile Hemangiomas: Usability Study. Journal of Medical Internet Research, 15(12), 1-1. doi: 10.2196/resprot.2897	Health issue
Hallett, J., Maycock, B., Kypri, K., Howat, P., & Mcmanus, A. (2009). Development of a Web-based alcohol intervention for university students: Processes and challenges. Drug & Alcohol Review, 28(1), 31-39. doi: 10.1111/j.1465-3362.2008.00008.x	Not users' psychological experience
Hightow-Weidman, L. B., Fowler, B., Kibe, J., McCoy, R., Pike, E., Calabri, M., & Adimora, A. (2011). HealthMpowerment.org: Development of a Theory-Based HIV/STI Website for Young Black MSM. AIDS Education & Prevention, 23(1), 1-12. doi: 10.1521/aeap.2011.23.1.1	Not users' psychological Experience
Houston, T. K., & Ford, D. E. (2008). A tailored Internet-delivered intervention for smoking cessation designed to encourage social support and treatment seeking: usability testing and user tracing. Informatics for Health & Social Care, 33(1), 5-19.	Not users' psychological Experience
Jelin, E., Granum, V., & Eide, H. (2012). Experiences of a Web-Based Nursing Intervention—Interviews with Women with Chronic Musculoskeletal Pain. Pain Management Nursing, 13(1), 2-10. doi: 10.1016/j.pmn.2011.08.008	Intervention type
Kelders, S. M., van Gemert-Pijnen, J. E., Werkman, A., & Seydel, E. R. (2010). Evaluation of a web-based lifestyle coach designed to maintain a healthy bodyweight. Journal of Telemedicine & Telecare, 16(1), 3-7. doi: 10.1258/jtt.2009.001003	Health issue
Kelders, S. M., Pots, W. T., Oskam, M. J., Bohlmeijer, E. T., & van Gemert-Pijnen, J. E. (2013). Development of a web-based intervention for the indicated prevention of depression. BMC Medical Informatics And Decision Making, 13, 26-26. doi: 10.1186/1472-6947-13-26	Usability focus only
Kerr, C., Murray, E., Burns, J., Turner, I., Westwood, M.A., Macadam, C., Nazareth, I., & Patterson, D. (2008). Applying user-generated quality criteria to develop an Internet intervention for patients with heart disease. Journal of telemedicine and telecare, 14(3):124-7. doi: 10.1258/jtt.2008.003006.	Health issue
Latalova, K., Prasko, J., Kamaradova, D., Jelenova, D., Ociskova, M., & Sedlackova, Z. (2014). Internet Psychoeducation for Bipolar Affective Disorder: Basis for Preparation and First Experiences. Psychiatric Quarterly, 85(2), 241-255. doi: 10.1007/s11126-013-9286-y	Intervention type

Reference	Reason to exclude
Luger, T. M., Houston, T. K., & Suls, J. (2014). Older adult experience of online diagnosis: results from a scenario-based think-aloud protocol. Journal of Medical Internet Research, 16(1), e16-e16. doi: 10.2196/jmir.2924	Intervention type
Lyles, C. R., Harris, L. T., Le, T., Flowers, J., Tufano, J., Britt, D., . . . Ralston, J. D. (2011). Qualitative evaluation of a mobile phone and web-based collaborative care intervention for patients with type 2 diabetes. Diabetes Technology & Therapeutics, 13(5), 563-569. doi: 10.1089/dia.2010.0200	Health issue
Maierle, D., & Ryan, P. (2011). Evaluation of satisfaction and use of electronic intervention for behavior change. CIN: Computers, Informatics, Nursing, 29(11), 622-629. doi: 10.1097/ncn.0b013e318224b4b2	Not users' psychological Experience
Markham, C. M., Shegog, R., Leonard, A., Bui, T. C., & Paul, M. E. (2009). +CLICK: harnessing web-based training to reduce secondary transmission among HIV-positive youth. AIDS Care, 21(5), 622-631. doi: 10.1080/09540120802385637	Not users' psychological Experience
Marziali, E., & Garcia, L. J. (2011). Dementia caregivers' responses to 2 internet-based intervention programs. American Journal of Alzheimer's Disease & Other Dementias, 26(1), 36-43. doi: 10.1177/1533317510387586	Intervention type
McTigue, K. M., Bhargava, T., Bryce, C. L., Conroy, M., Fischer, G. S., Hess, R., . . . Zickmund, S. (2011). Patient perspectives on the integration of an intensive online behavioral weight loss intervention into primary care. Patient Education & Counseling, 83(2), 261-264. doi: 10.1016/j.pec.2010.05.009	Health issue
Meglic, M., Furlan, M., Kuzmanic, M., Kozel, D., Baraga, D., Kuhar, I., . . . Brodnik, A. (2010). Feasibility of an eHealth service to support collaborative depression care: results of a pilot study. Journal of Medical Internet Research, 12(5), e63-e63. doi: 10.2196/jmir.1510	Not users' psychological Experience
Ossebaard, H. C., Seydel, E. R., & van Gemert-Pijnen, L. (2012). Online usability and patients with long-term conditions: a mixed-methods approach. International Journal of Medical Informatics, 81(6), 374-387. doi: 10.1016/j.ijmedinf.2011.12.010	Health issue
Riiser, K., Løndal, K., Ommundsen, Y., Sundar, T., & Helseth, S. (2013). Development and Usability Testing of an Internet Intervention to Increase Physical Activity in Overweight Adolescents. Journal of Medical Internet Research, 15(1), 7-7. doi: 10.2196/resprot.2410	Not users' psychological experience and health issue
Ruggiero, L., Moadsiri, A., Quinn, L. T., Riley, B. B., Danielson, K. K., Monahan, C., . . . Gerber, B. S. (2014). Diabetes Island: Preliminary Impact of a Virtual World Self-Care Educational Intervention for African Americans With Type 2 Diabetes. Journal of Medical Internet Research, 16(8), 1-1. doi: 10.2196/games.3260	Health issue
Ryan, P., Pumilia, N. J., Henak, B., & Chang, T. (2009). Development and performance usability testing of a theory-based, computerized, tailored intervention. CIN: Computers, Informatics, Nursing, 27(5), 288-300. doi: 10.1097/NCN.0b013e3181b21779	Not users' psychological experience and health issue

**Reference**	**Reason to exclude**
Saulsberry, A., Corden, M., Taylor-Crawford, K., Crawford, T., Johnson, M., Froemel, J., . . . Van Voorhees, B. (2013). Chicago Urban Resiliency Building (CURB): An Internet-Based Depression-Prevention Intervention for Urban African-American and Latino Adolescents. Journal of Child & Family Studies, 22(1), 150-160. doi: 10.1007/s10826-012-9627-8	Not users' psychological Experience
Shegog, R., Markham, C. M., Leonard, A. D., Bui, T. C., & Paul, M. E. (2012). "+CLICK": pilot of a web-based training program to enhance ART adherence among HIV-positive youth. AIDS Care, 24(3), 310-318. doi: 10.1080/09540121.2011.608788	Health issue and not users' psychological Experience
Swan, A. J., & Tyssen, E. G. (2009). Enhancing treatment access: Evaluation of an Australian Web-based alcohol and drug counselling initiative. Drug & Alcohol Review, 28(1), 48-53. doi: 10.1111/j.1465-3362.2008.00006.x	Intervention type
Van Voorhees, B. W., Gollan, J., & Fogel, J. (2012). Pilot study of Internet-based early intervention for combat-related mental distress. Journal of Rehabilitation Research & Development, 49(8), 1175-1190. doi: 10.1682/jrrd.2011.05.0095	Not users' psychological experience
Voncken-Brewster, V., Moser, A., van der Weijden, T., Nagykaldi, Z., de Vries, H., & Tange, H. (2013). Usability evaluation of an online, tailored self-management intervention for chronic obstructive pulmonary disease patients incorporating behavior change techniques. JMIR Research Protocols, 2(1), e3-e3. doi: 10.2196/resprot.2246	Not users' psychological experience and health issue
Williams, R. A., Gatien, G., & Hagerty, B. (2011). Design element alternatives for stress-management intervention websites. Nursing Outlook, 59(5), 286-291.e283. doi: 10.1016/j.outlook.2011.03.009	Not users' psychological experience
Williams, S., Yardley, L., & Wills, G. B. (2013). A qualitative case study of LifeGuide: Users' experiences of software for developing Internet-based behaviour change interventions. Health Informatics Journal, 19(1), 61-75. doi: 10.1177/1460458212458915	Intervention type
